# MYC-Driven Pathways in Breast Cancer Subtypes

**DOI:** 10.3390/biom7030053

**Published:** 2017-07-11

**Authors:** Yassi Fallah, Janetta Brundage, Paul Allegakoen, Ayesha N. Shajahan-Haq

**Affiliations:** Department of Oncology, Lombardi Comprehensive Cancer Center, Georgetown University Medical Center, Washington, DC 20057, USA; yf120@georgetown.edu (Y.F.); janetta@rsjbrun.com (J.B.); pna8@georgetown.edu (P.A.)

**Keywords:** breast cancer, ER, HER2, TNBC, drug resistance

## Abstract

The transcription factor MYC (MYC proto-oncogene, bHLH transcription factor) is an essential signaling hub in multiple cellular processes that sustain growth of many types of cancers. MYC regulates expression of RNA, both protein and non-coding, that control central metabolic pathways, cell death, proliferation, differentiation, stress pathways, and mechanisms of drug resistance. Activation of MYC has been widely reported in breast cancer progression. Breast cancer is a complex heterogeneous disease and treatment options are primarily guided by histological and biochemical evaluations of the tumors. Based on biochemical markers, three main breast cancer categories are ER+ (estrogen receptor alpha positive), HER2+ (human epidermal growth factor receptor 2 positive), and TNBC (triple-negative breast cancer; estrogen receptor negative, progesterone receptor negative, HER2 negative). MYC is elevated in TNBC compared with other cancer subtypes. Interestingly, MYC-driven pathways are further elevated in aggressive breast cancer cells and tumors that display drug resistant phenotype. Identification of MYC target genes is essential in isolating signaling pathways that drive tumor development. In this review, we address the role of MYC in the three major breast cancer subtypes and highlight the most promising leads to target MYC functions.

## 1. Introduction

MYC (MYC proto-oncogene, bHLH transcription factor) expression is deregulated in various cancer types. In breast cancer, MYC is overexpressed in 30–50% of high-grade tumors [1,2]. As a transcription factor, MYC exhibits site-specific DNA-binding activity with its binding factor MYC associated factor X (MAX). This MYC–MAX binding is rate-limiting for cell cycle progression through G1 phase and this process is partly regulated by cyclin-dependent kinases [3,4] in cell proliferation. In addition, MYC plays critical roles in multiple cellular pathways that promotes survival of cancer cells [5,6]. MYC plays important roles in the pathogenesis of cancer and is particularly important in the survival of cancer cells that are resistant to anti-cancer drugs. Thus, targeting MYC is a logical strategy in drug resistant breast cancers. However, due to the lack of pharmacological efficacy of direct MYC inhibitors [7], to circumvent the issue, researchers have shifted their focus on understanding the target genes and pathways downstream of MYC activation. In this review, we focus on the role of MYC in the three main breast cancer subtypes and the MYC-driven pathways that can be targeted in the clinic. 

## 2. Breast Cancer

Over 1 million new breast cancer cases are diagnosed each year worldwide and account for most cancer-related deaths in women [8]. With multiple subtypes and molecular markers, breast cancer is a heterogeneous disease and successful treatment in the clinic is hampered by reliable biomarkers [9]. For localized breast cancer, the standard of care involves lumpectomy or mastectomy with or without radiation, while systemic adjuvant therapies (chemotherapy, endocrine therapy or biologic therapy) is used to improve survival [10]. 

Biochemical features of tumors—such as hormone receptor status including estrogen receptor alpha (ER), progesterone receptor (PR) or growth factor receptor status such as human epidermal growth factor receptor 2 (HER2) positive expression or histological grade—are determined by immuno-histochemical stains (IHC) [11]. Furthermore, overall, clinical considerations including age, menopausal status, lymph node invasion, and tumor size are also essential in determining the best therapeutic option for a breast cancer patient. Nevertheless, the ability of breast cancer cells to circumvent drug mediated cell death, i.e., drug resistance, is common in all types of breast cancer and still remains an unsolved clinical problem. In addition, the levels of ER and biochemical profile can change as the cancer progresses or as it is treated with different therapies [12,13]. The oncoprotein MYC is a master regulator of many cellular signaling and metabolic pathways and has been implicated in drug resistance in breast cancer by allowing cancer cells to reprogram under specific drug induced stress [14]. Here, we discuss some of the findings that focus on the role of MYC in promoting cancer progression in three of the major breast cancer subtypes. 

## 3. ER positive Breast Cancer

Majority (~70%) of breast cancer tumors are ER positive (ER+) [15] and are treated with endocrine therapy that block ER activity with antiestrogens such as Tamoxifen or Faslodex/Fulvustrant (ICI 182, 780) [16,17,18] or diminish estrogen-mediated signaling by reducing estrogen synthesis with aromatase inhibitors [19]. While endocrine therapy is effective in treating a vast majority of ER+ tumors, about 50% of all ER+ breast cancer cases will not respond (de novo resistance) or gain resistance (acquired resistance) [18]. While the precise molecular mechanism for endocrine resistance remains unclear, emerging data suggests that MYC overexpression may contribute to acquired resistance in ER+ breast cancers. 

MYC is an estrogen responsive gene [20] and its overexpression is implicated in hormone-independence in ER+ breast cancer cell and tumor models [21,22,23]. Estrogen-mediated induction of *myc* gene expression is via an upstream enhancer activated by ER and activator protein 1 (AP-1) transcription factor [24]. In human tumors, *myc* overexpression has been linked to resistance to endocrine therapies [22,25]. Increased *myc* expression has been observed in estrogen-independent derivatives of MCF-7 cells, an ER+ breast cancer cell model [21,25,26]. In order to understand how MYC confers endocrine resistance in ER+ breast cancer, the downstream effectors of MYC that regulates cell survival needs to be determined. It has been shown that high mRNA levels of HSPC111(HBV pre-S2 trans-regulated protein 3) correlated with MYC mRNA levels and is associated with an adverse patient outcome. HSPC111 has been identified as an estrogen-responsive gene in breast cancer cell models [27] and this multi-protein complex plays a crucial role in ribosomal biogenesis and protein production [28]. Additionally, the role of MYC in endocrine resistance may be linked to its function in cellular metabolism, particularly in glutaminolysis and glycolysis [21,23]. ER+ breast cancer cells that are resistant to endocrine therapy such as Faslodex and Tamoxifen, overexpress MYC and are better adapted to withstand periods of glucose deprivation. Moreover, these MYC overexpressing breast cancer cells can use glutamine to support cell survival through the unfolded protein response (UPR) [21]. In ER+ breast cancer cells with an aromatase inhibitor resistant phenotype, MYC expression is upregulated by the cross-talk between ER and HER2 and Faslodex inhibited MYC, glutamine transporter solute carrier family (SLC) 1A5, glutaminase (GLS), and glutamine consumption [23]. 

## 4. HER2-positive Breast Cancer

HER2 is overexpressed in 20% of breast cancers primarily due to *HER2* gene amplification. HER2 breast cancer is associated with increased proliferative indices, metastasis and recurrence [29]. HER2-targeted therapy includes the anti-HER2 targeting recombinant monoclonal antibody (e.g., Trastuzumab/Herceptin or Prtuzimab/Perjeta) and adenosine triphosphate (ATP)-competitive tyrosine kinase inhibitors (TKIs; e.g., Lapatinib/Tykerb) or an antibody-drug conjugate (Trastuzumab-Emtansine; T-DM1/Kadcyla) that combines Tastuzumab with cytotoxic agent, Emtansine [30]. While Trastuzumab has significantly improved outcome in breast cancer patients, including prolonged progression-free periods and overall survival, a large number (40–60%) of HER2 positive (HER2+) tumors will develop de novo or acquired resistance to Trastuzumab [31]. 

While TKIs provide clinical benefit to a subset of patients (<25%) progressing on Trastuzumab, most patients develop resistance to the TKIs [32]. Therefore, resistance to these targeted therapies remains a major clinical issue in treating HER2+ breast cancers and understanding mechanisms of drug action is imperative in developing more effective treatment options. MYC gene copy number gain may adversely impact Trastuzumab treated metastatic breast cancer patient outcome [33]. In sensitive breast cancer cell models, Trastuzumab and Lapatinib blocked extracellular signal-regulated kinases 1/2 and phosphatidylinositol-3 kinase (PI3K)/AKT that in turn inhibited MYC activation and upregulated miR-16, a novel tumor suppressor [34]. A subset of breast cancers that are ER+/HER2+ may not benefit from TKIs, possibly due to bidirectional cross-talk between ER and HER2. In vitro studies have shown that when growth factor receptor signaling is dominant, Trastuzumab can lead to repression of MYC activation, however, this inhibition is ineffective then ER signaling pathway is active [35]. 

## 5. Triple-Negative Breast Cancer

Triple-Negative Breast Cancer (TNBC) constitutes about 10% of all breast cancer cases and is a subtype of breast cancer without a defined drug target. In comparison to ER+ or HER2+ breast cancers, MYC expression is markedly elevated in TNBC [36] along with altered expression of MYC regulated genes that potentiates MYC regulated pathways. Since a direct target MYC remains a challenge [37], an alternate innovative approach to identify and target signaling pathways activated by MYC selectively in tumors referred to as synthetic lethality [38] has been explored in breast cancer. A synthetic lethal interaction between MYC overexpression and inhibition of cyclin-dependent kinase (CDK) has shown growth inhibition in TNBC xenografts [39]. MYC has been shown to regulate glutamine metabolism in multiple cancer cell types [6]. Interestingly, a small molecule inhibitor of glutaminase (GLS), CB-839, has been shown to inhibit growth of TNBC xenografts as a single agent and in combination with paclitaxel [40], and therefore, CB-839 may be an effective as a part of a combination anti-cancer therapy for TNBC cases in the clinic. 

While MYC overexpression can have a dominant impact on a host of genes that promote tumor growth, combination of MYC overexpression with concurrent loss or activation of other genes may contribute to aberrant tumor growth in TNBC. Amplification of MYC with loss of tumor suppressor pathways such as p53 and RB (retinoblastoma), can promote a particularly poor outcome in TNBC [41]. MYC overexpression correlated with poor prognosis in sporadic breast cancer with susceptibility gene 1 (BRCA1) deficiency with a TNBC phenotype [42], and thus, a combination therapy targeting BRCA1 deficiency and the MYC pathway may provide a promising strategy for this specific subtype of breast cancer. In MYC-overexpressing TNBC patient-derived xenografts, researchers have used metabolomics analysis to identify a lipid metabolism gene signature and they have shown that pharmacological inhibition of fatty acid oxidation (FAO) can decrease tumor growth [43]. Therefore, FAO inhibitors could be a potential therapeutic strategy for MYC overexpressing TNBC tumors. MYC drives glucose metabolism in TNBC cells by directly binding an E-box-containing region in the promoter and repressed thioredoxin-interacting protein (TXNIP), a potent negative regulator of glucose uptake and glycolysis. A high-MYC and low-TXNIP gene signature correlates with decreased overall survival and decreased metastasis-free survival in TNBC. Furthermore, mutation of TP53, a frequent occurrence in TNBC, enhances the correlation between the high-MYC and low-TXNIP gene signature and death from breast cancer [44]. 

## 6. Conclusions

MYC amplification is present in 30–50% of high-grade breast cancers. MYC-dependent pathways are often elevated in acquired resistance to anti-cancer therapies. Therefore, targeting MYC effectors may be a useful strategy in treating drug resistant, MYC-dependent tumors. Additionally, MYC amplification can be used as a useful predictive marker for drug resistance in breast cancer. Regardless of breast cancer subtypes, MYC amplification has been reported to be a predictive factor of complete pathologic response to neoadjuvant chemotherapy (Anthracycline-Cyclophosphamide followed by Taxane Docetaxel; AC + T) regimen [45]. Considering the essential role that MYC plays in promoting cell proliferation in breast cancer, it is imperative to determine external or environmental factors that regulate MYC expression in breast cancer cells. For example, xenoestrogens, synthetic compounds that mimic endogenous estrogens, such as parabens, which are used in personal care products, has been shown to activate MYC mRNA in ER−/HER2+ breast cancer cell models through cross-talk between ER and HER2 signaling [46]. Whether exposure to such xenografts exacerbates anti-cancer drug resistance in breast cancer remains unknown. Thus, knowledge of MYC-driven networks in breast cancer progression will expand our range of pharmacological inhibitors that can kill cancer cells with precision and efficacy. 
